# Gender trends in match rate to surgical specialties in Canada: A retrospective study from 2003–2022

**DOI:** 10.1371/journal.pone.0300207

**Published:** 2024-04-10

**Authors:** Mostafa Bondok, Mohamed S. Bondok, Anne Xuan-Lan Nguyen, Christine Law, Nawaaz Nathoo, Nupura Bakshi, Nina Ahuja, Karim F. Damji

**Affiliations:** 1 Faculty of Medicine, University of British Columbia, Vancouver, BC, Canada; 2 Cumming School of Medicine, University of Calgary, Calgary, AB, Canada; 3 Faculty of Medicine and Health Sciences, McGill University, Montreal, QC, Canada; 4 Department of Ophthalmology, School of Medicine, Queen’s University, Kingston, ON, Canada; 5 Department of Ophthalmology and Visual Sciences, Faculty of Medicine, University of British Columbia, Vancouver, BC, Canada; 6 Department of Ophthalmology and Vision Sciences, Temerty Faculty of Medicine, Toronto, ON, Canada; 7 Division of Ophthalmology, Department of Surgery, McMaster University, Hamilton, ON, Canada; 8 Department of Ophthalmology and Visual Sciences, Aga Khan University, Karachi, Pakistan; 9 Department of Ophthalmology and Visual Sciences, University of Alberta, Edmonton, AB, Canada; University of Oxford, UNITED KINGDOM

## Abstract

**Background:**

In Canada, there is a recognized underrepresentation of women in the field of surgery. However, the extent to which this trend applies across various surgical specialties is not well delineated. The aim of this study is to identify existing disparities and trends over time to inform the need for future interventions to make the match process more equitable for applicants.

**Methods:**

Data regarding surgical specialty applicants was extracted from the Canadian Resident Matching Service (CaRMS)’s 2003 to 2022 reports.

**Results:**

A total of 9,488 applicants ranked surgical specialties as their first choice from 2003–2022. Increases in the proportion of women applicants comparing periods 2003–2007 to 2018–2022 were significant for cardiac surgery (22% to 43%, p = 0.03), general surgery (46% to 60%, p<0.001), orthopedic surgery (23% to 35%, p<0.001), urology (23% to 38%, p<0.001), and all aggregated surgical specialties (‘all surgery’) (45% to 55%, p<0.001). An increase in the proportion of women applicants who matched over the same periods was observed for general surgery (+47% to 60%, p<0.001), orthopedic surgery (24% to 35%, p<0.01), urology (21% to 34%, p<0.001), and all surgery (46% to 54%, p<0.001). From 2003–2022, a lower match rate for women compared to men was observed for otolaryngology (0.60 v 0.69, p = 0.008), urology (0.61 v 0.72, p = 0.003), and all surgery (0.71 v 0.73, p = 0.038), while higher match rates were observed for ophthalmology (0.65 v 0.58, p = 0.04). No statistically significant differences in match rate were observed from 2018–2022.

**Conclusions:**

While the proportion of women applicants to surgical specialties in Canada has been increasing, women remain underrepresented in several surgical specialties. This underrepresentation cannot be solely attributed to fewer women applying to these specialties, as women experience lower success rates when matching to specific surgical specialties. Further research is essential to identify and address the underlying causes of these disparities.

## Introduction

The Canadian Resident Matching Service (CaRMS) is an algorithm-based matching service which facilitates the application and selection process for medical students applying to various residency programs across Canada [1]. This independent, non-profit organization aims to be an equitable merit-based selection process [1]. In response to concerns regarding the transparency in the Canadian residency selection process, the Best Practices in Applications and Selection (BPAS) report outlines recommendations to ensure fairness in the residency selection process, including promoting diversity in the resident body [2]. The report further emphasizes the need to increase gender diversity in specialties that have been traditionally skewed towards a single gender [2]. Indeed, while the number of women residents and physicians in Canada increases, the match rate of women to surgical specialties does not reflect this trend and tends towards higher match rates for men [3]. This disparity may impact perceived or actual mentorship opportunities for women interested in surgery [4–7].

Studies utilizing CaRMS data have shown differing outcomes in match results for men and women applicants. These differences can, in part, be attributed to variations in research methodologies including outcome measures, the specific surgical specialties examined, and the time periods examined in the studies [3, 8–12]. This makes direct comparisons between surgical specialties challenging, limiting opportunities for cross-disciplinary learning between specialties. For instance, a 10-year analysis of Canadian urology residency programs using CaRMS data from 2007–2017 found no significant differences in match rate to urology by gender [8]. However, a subsequent study using CaRMS data from 2013–2019 found women less likely to match to urology [12]. Lorello et al., found that women applying to surgery were less likely to match using CaRMS data [10], however, the magnitude of this difference and whether this is true for individual surgical specialties was not explored.

Current underrepresentation of women in the surgical field underscores the need for a contemporary and comprehensive analysis to assess the extent of this gender disparity across all surgical specialties during residency training, and to investigate whether differences in applicant success by gender contribute to this phenomenon. In this study, we examine all surgical disciplines over the past two decades, to determine whether any specialty-specific underrepresentation is a result of disparities in gender-specific match rates (where women applying face lower acceptance rates) or from a lower proportion of women applicants to select surgical specialties. By identifying existing disparities and progress made over time, this may provide stakeholders with benchmarks, and inform the need for future interventions to make the match process more equitable for applicants.

## Methods

A cross-sectional review of Canadian Medical Graduates (CMGs) who applied (“applicants”) and applicants who matched (“matriculants”) to surgical disciplines through CaRMS was conducted. CMGs refers to applicants from both Canada and the United States. This study was exempted from requiring ethics approval by the University of British Columbia Behavioural Research Ethics Broad (BREB). Data on first choice applicants and matriculants by gender was extracted from the previous 20 match cycles on the CaRMS “Data and reports” web page, spanning the period from 2003–2022.

This study had three outcomes of interest: the proportion of applicants by gender, the proportion of matriculants by gender, and match rate by gender. Match rate was computed as matriculants divided by applicants, for men and women respectively. Four-year intervals were used to overcome the weak statistical power conferred by a year-over-year analysis of small surgical training programs in Canada [13]. The intervals assessed were: 2003–2007, 2008–2012, 2013–2017, and 2018–2022.

All surgical disciplines were analyzed, including cardiac surgery, general surgery, neurosurgery, obstetrics and gynecology, ophthalmology, orthopedic surgery, otolaryngology, plastic surgery, urology, and vascular surgery. The term ‘all surgery’ is used to denote the aggregate findings of all surgical disciplines. First choice applicants to clinical investigator or research track surgical-based positions were excluded as these positions were not offered consistently over the study period.

Gender trends were analyzed using central (mean) and dispersion values (standard deviation), in addition to testing for gender proportions. Fisher’s exact test was used to individually assess the association between matching to a first choice surgical discipline and gender, averaged over the four-year intervals noted above. The Cochrane-Armitage trend test for proportions was used to assess trends in the proportion of first choice women applicants and matriculants as well as the overall match rate of men and women applicants to surgical specialties over the intervals. Statistical analyses were conducted using IBM SPSS Statistics Version 27 (IBM Corporation, Armonk, New York) and R Version 4.2.1. All tests were two-tailed, and P values less than 0.05 were considered statistically significant.

## Results

A total of 9,488 applicants ranked surgical specialties as their first choice from 2003–2022. This includes cardiac surgery (n = 194), general surgery (n = 1,935), neurosurgery (n = 409), obstetrics and gynecology (n = 2,044), ophthalmology (n = 1,077), orthopedic surgery (n = 1,260), otolaryngology (n = 772), plastic surgery (n = 891), urology (n = 784), and vascular surgery (n = 122).

The proportion of women graduates from Canadian medical schools has exceeded that of men graduates during all four-year intervals assessed (S1 Fig). There were 55.4% (4349/7857) women in 2003–2007 compared to 57.4% (6935/12085) in 2008–2012, 55.7% (7908/14201) in 2013–2017, and 55.3% (8037/14521) in 2018–2022. No significant trends were observed between these four intervals (p = 0.15) (S1 Fig).

**Fig 1:**
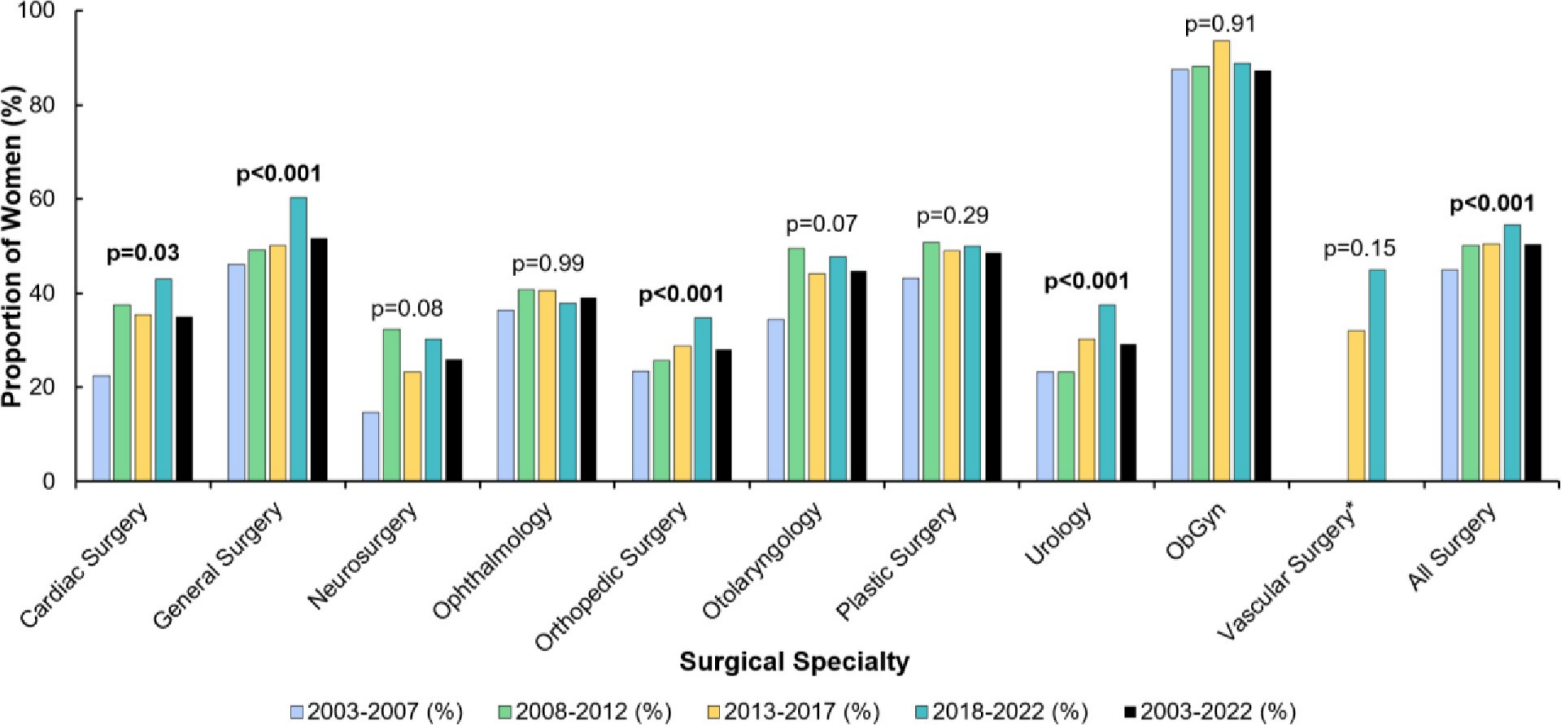
Proportion of women applicants to surgical specialties from 2003–2022. *Data for vascular surgery not available prior to 2012. P values are from the Cochran-Armitage trend test for proportions. Bold font indicates statistical significance (p<0.05). The formula used for percentage of first choice women applicants was (women applicants / [men applicants + women applicants]) *100. ObGyn: Obstetrics and Gynecology.

All surgical specialties exhibited an increase in the proportion of women applicants, but not all were significant (Fig 1). Significant increases in the proportion of women applicants from the 2003–2007 interval to the 2018–2022 interval were present for cardiac surgery (22% [11/49] to 43% [28/65], p = 0.03), general surgery (46% [171/371] to 60% [302/500], p<0.001), orthopedic surgery (23% [64/273] to 35% [99/284], p<0.001), urology (23% [35/150] to 38% [83/221], p<0.001), and all surgery (45% [810/1801] to 55% [1426/2615], p<0.001). Tabulated values are available for reference (S1 Table).

**Fig 2:**
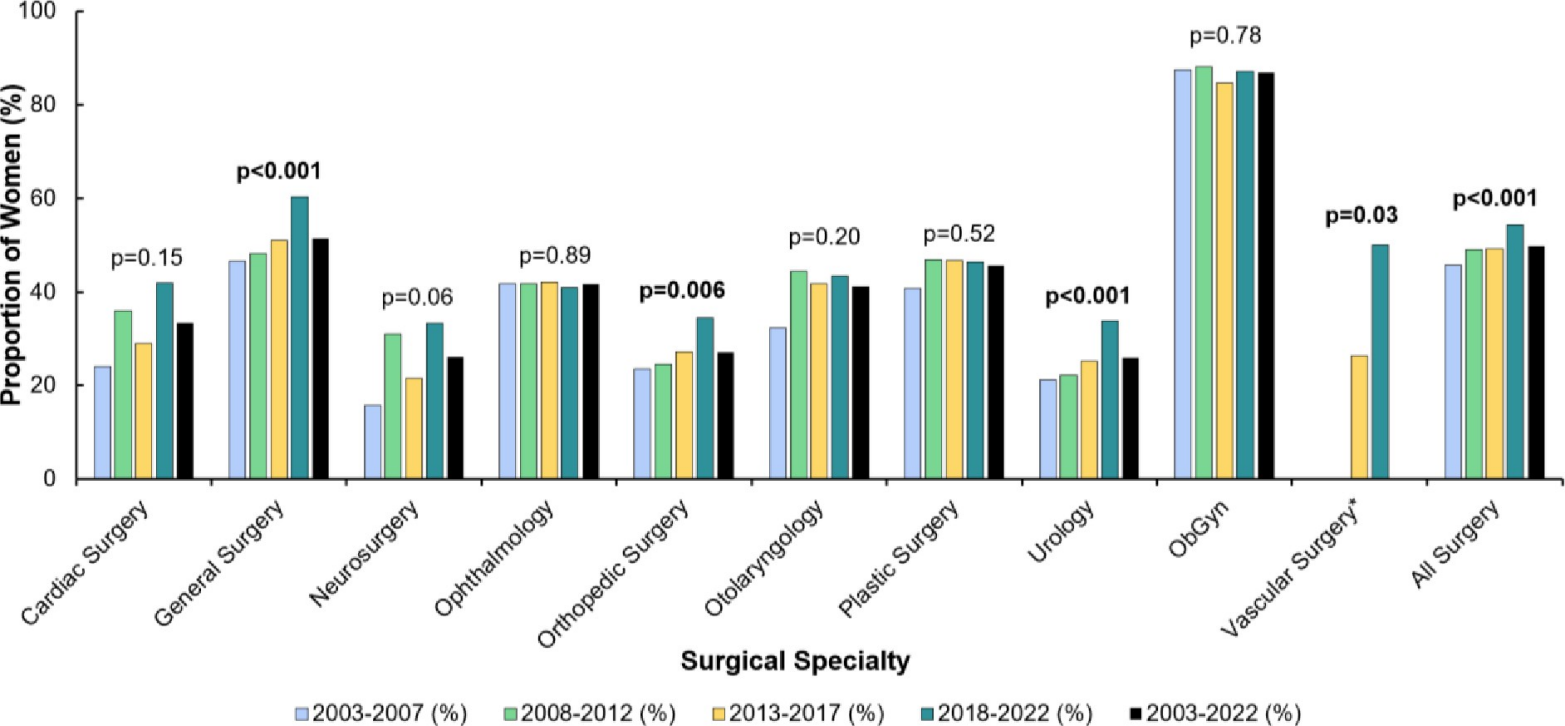
Proportion of women matriculants to surgical specialties from 2003–2022. *Data for vascular surgery not available prior to 2012. P values are from the Cochran-Armitage trend test for proportions. Bold font indicates statistical significance (p<0.05). The formula used for percentage of first choice women matriculants was (women matriculants / [men matriculants + women matriculants]) *100. ObGyn: Obstetrics & Gynecology.

Fig 2 illustrates a significant increase in the proportion of women matriculants from the 2003–2007 period to the 2018–2022 period for general surgery (47% [153/329] to 60% [217/360], p<0.001), orthopedic surgery (24% [55/233] to 35% [77/223], p<0.01), urology (21% [22/82] to 34% [48/142], p<0.001), and all surgery (46% [616/1348] to 54% [928/1705], p<0.001). A significant increase in the proportion of first choice women matriculants from the 2013–2017 to the 2018–2022 period was present for vascular surgery (26% [10/38] to 50% [20/40], p = 0.03). Tabulated values are available for reference (S2 Table).

Accumulated over the 20-year study period, Table 1 demonstrates a lower match rate for women applicants compared to men applicants was observed for otolaryngology (women: 0.60 [207/345] vs men: 0.69 [296/427], p = 0.008), urology (women: 0.61 [139/229] vs men: 0.72 [398/555], p = 0.003), and all surgery (women: 0.71 [3386/4789] vs men: 0.73 [3413/ 4699], p = 0.038). Ophthalmology was the only surgical specialty with a higher match rate for women relative to men over the 20-year interval (women: 0.65 [273/421] vs men: 0.58 [382/656], p = 0.04) (Table 1). Significant decreases in match rate for women applicants from the 2003–2007 interval to the 2018–2022 interval were present for general surgery (0.89 [153/171] to 0.72 [217/302], p<0.001) and obstetrics and gynecology (0.80 [258/323] to 0.68 [335/493], p<0.001), while decreases in match rate for men applicants were present for general surgery (0.88 [176/200] to 0.72 [143/198], p<0.001) and neurosurgery (0.84 [176/200] to 0.70 [143/198], p = 0.01). A significant decrease in match rate for men applicants from the 2013–2017 interval to the 2018–2022 interval was present for vascular surgery (0.78 [28/36] to 0.53 [20/38], p = 0.02) (Table 1).

**Table 1:** Trends and gender differences in match rates for applicants to surgical specialties from 2003–2022.

Specialty	2003–2007			2008–2012			2013–2017			2018–2022			2003–2022			Trend p value^‡^	
	Women	Men	p*	Women	Men	p*	Women	Men	p*	Women	Men	p*	Women	Men	p*	Women	Men
**Cardiac Surgery**	0.73	0.66	1.00	0.83	0.90	0.62	0.59	0.81	0.17	0.68	0.70	1.00	0.69	0.75	0.50	0.49	0.73
**General Surgery**	0.89	0.88	0.74	0.80	0.84	0.32	0.78	0.75	0.47	0.72	0.72	1.00	0.79	0.80	0.58	**<0.001**	**<0.001**
**Neurosurgery**	0.92	0.84	0.68	0.79	0.85	0.58	0.67	0.73	0.61	0.81	0.70	0.27	0.78	0.78	1.00	0.53	**0.01**
**Ophthalmology**	0.70	0.56	0.08	0.63	0.60	0.71	0.74	0.69	0.49	0.57	0.50	0.22	0.65	0.58	**0.04**	0.19	0.29
**Orthopedics**	0.86	0.85	1.00	0.82	0.87	0.19	0.81	0.88	0.20	0.78	0.79	0.88	0.81	0.85	0.10	0.20	0.11
**Otolaryngology**	0.59	0.65	0.48	0.65	0.80	**0.03**	0.64	0.71	0.37	0.52	0.62	0.14	0.60	0.69	**0.008**	0.22	0.35
**Plastic Surgery**	0.43	0.48	0.55	0.50	0.59	0.19	0.47	0.52	0.53	0.45	0.52	0.36	0.47	0.53	0.08	0.95	0.88
**Urology**	0.63	0.71	0.40	0.70	0.74	0.58	0.57	0.73	**0.03**	0.58	0.68	0.15	0.61	0.72	**0.003**	0.32	0.54
**ObGyn**	0.80	0.80	1.00	0.80	0.80	1.00	0.72	0.72	1.00	0.68	0.79	0.08	0.74	0.77	0.36	**<0.001**	0.61
**Vascular^†^**	-	-	-	-	-	-	0.59	0.78	0.20	0.65	0.53	0.34	-	-	-	0.70	**0.02**
**All Surgery**	0.76	0.74	0.30	0.74	0.78	0.058	0.70	0.73	**0.046**	0.65	0.65	0.90	0.71	0.73	**0.038**	**<0.001**	**<0.001**

*P values were determined using fisher’s exact test.

†Data for vascular surgery not available prior to 2012.

‡Trend P values are from the Cochran-Armitage trend test for proportions. Bold font indicates statistical significance (p<0.05). The formula used for match rate for first choice women and men applicants was (women matriculants / women applicants), and (men matriculants / men applicants), respectively. ObGyn: Obstetrics and Gynecology. Orthopedics: Orthopedic Surgery.

**Fig 3:**
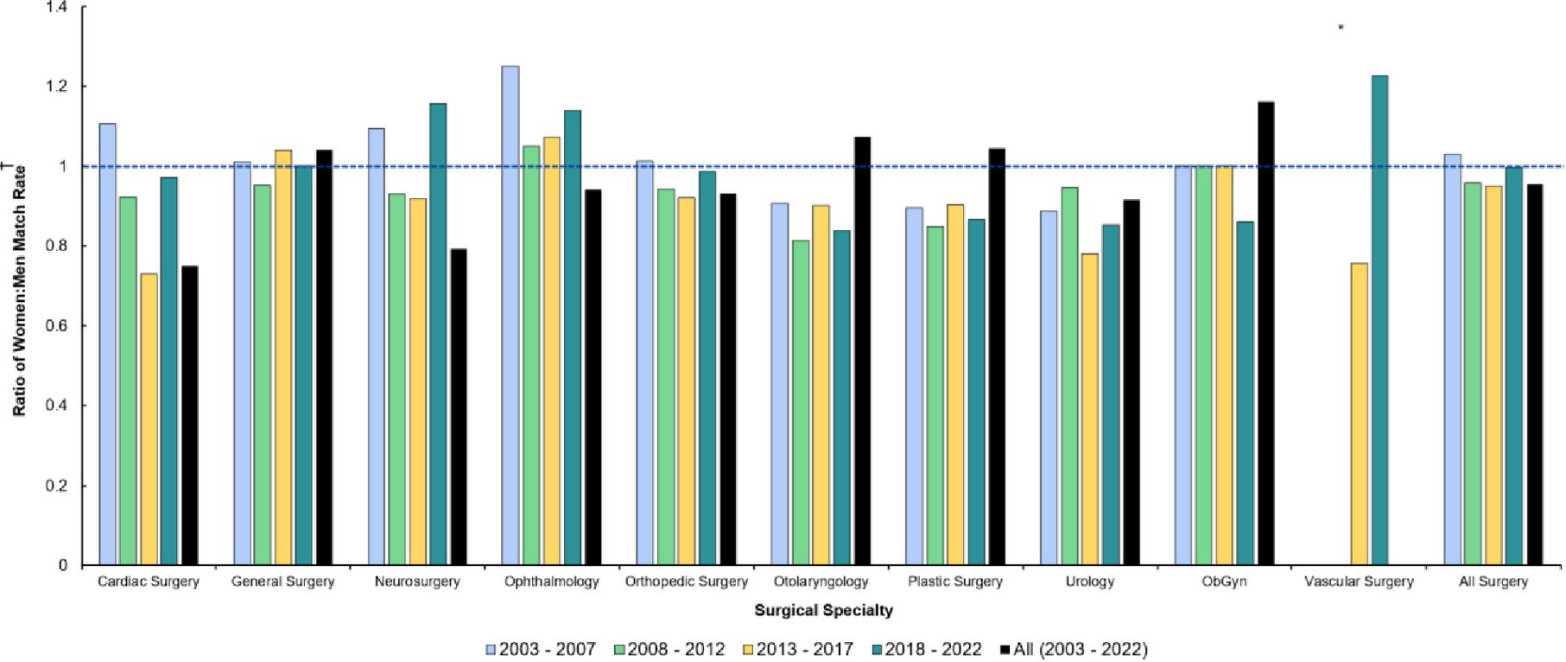
Ratio between women and men match rate to surgical specialties from 2003–2022. *Data for vascular surgery not available prior to 2012. †The formula used for ratio of women to men match rate was: (women match rate / men match rate) = ([women matriculants / women applicants] / [men matriculants / men applicants]). ObGyn: Obstetrics and Gynecology.

Fig 3 provides a visual representation of Table 1, but as a ratio of the match rate of women applicants, relative to men applicants. A value less than one (i.e., below the dotted blue line) indicates that the match rate for women applicants is lower than that of men applicants. The opposite is true if a value greater than one is demonstrated.

## Discussion

While the proportion of women graduates from Canadian medical schools has been higher than that of men graduates over the study period, women are underrepresented in most surgical specialties. This is partially due to the lower match success that women applying surgical specialties have compared to men, over this 20-year study period. However, statistically significant differences were only observed for otolaryngology, urology, and all surgery. This is not exclusively a phenomenon of the past. From 2018–2022, while not statistically significant, gender-based differences in match rate to several surgical specialties persist. For example, while women remain underrepresented in otolaryngology, plastic surgery, and urology from 2018–2022, their success rate of matching to these specialties are 19%, 16%, and 17% lower than men, respectively. The reasons for disparities in match success by gender warrant consideration by faculty and stakeholders involved in residency admissions. At the admissions level, discrimination policies for gender-based inequities [6, 14–16], and formal training to remediate against conscious and unconscious bias may help make the match process more equitable [4, 16].

Statistically significant increases in the proportion of women applicants were observed for cardiac surgery, general surgery, orthopedic surgery, urology, and all surgery. Meanwhile, statistically significant increases in the proportion of women matriculants were observed for vascular surgery, general surgery, orthopedic surgery, urology, and all surgery. Statistical significance may not a fair representation of progress within smaller programs, as the proportion of women matriculants in neurosurgery doubled, while that of cardiac surgery increased by 75%, but were not statistically significant.

To continue improving representation, efforts to attract more gender-diverse applicants to surgical specialties are needed. Deterrents from surgical specialty selection expressed by medical trainees include gender-related issues (e.g., unequal career opportunities, and unfair career advancement or leadership potential) [4, 6, 17–20], concerns regarding parental leave policies and incompatibility with having children and/or a family life [6, 19–21]. Indeed, disproportional rates of gender-based inequity or discrimination against surgical residents who are women have been documented [4, 14], while other studies have found women surgeons less likely to be married and have kids compared to their men colleagues [4, 5]. Strategies to increase the representation of women in surgery include early formal mentorship programs for students [4–6, 15, 16], compensation equity [4, 15, 16], adequate implementation of parental leave and childcare policies [4, 15].

Interestingly, the match rate of women applicants to obstetrics and gynecology has decreased over the last 4 years and is currently lower than that of men applicants. In contrast, the match of men applicants to neurosurgery has decreased over the study period and is now equal to that of women applicants. These findings contrast with existing literature, which suggests that men have more success in matching to specialties that are predominantly men, and vise versa, perhaps due to stereotyping, biases, and mentorship [20, 22–24]. These residency programs may have intentionally implemented such changes at the admissions level to increase gender representation, as obstetrics and gynecology is predominantly women (87%), while neurosurgery is predominantly men (74%).

The only surgical specialty with a statistically significant higher match rate for women applicants compared to men applicants over the study period was ophthalmology. In addition, the proportion of women matriculants to surgical specialties over the study periods analyzed has increased for all surgical specialties except ophthalmology, albeit not all these trends were significant.

Future studies exploring influences on medical student’s career choice (e.g., lifestyle, societal expectations, and mentorship opportunities), and gender-based factors that may contribute to inequities in the Canadian context may help explain the findings in this study. If made available in Canada as it has been in the United States [25, 26], data on demographic characteristics of applicants and residents (e.g., ethnicity, race, and geography) may inform strategic planning aimed at increasing recruitment of trainees to traditionally unpopular and/or underserved areas and improving the diversity of the surgical resident body.

The generalizability of this study is limited by several factors. First, match results of graduates from medical schools in Canada and the United States were analyzed, while international graduates were not. Second, the present study is limited in its ability to account for non-binary gender identities as CaRMS traditionally did not collect this information. Finally, while differences in match statistics were explored between men and women applicants to surgical disciplines, the consideration of other equity-relevant demographic characteristics such as race, non-binary gender identity, ethnicity, household income, or disability are important factors not analyzed due to limitations in data availability.

## Conclusion

While the proportion of women applicants to surgical specialties in Canada has been increasing across most surgical specialties, women remain underrepresented in several surgical specialties. This underrepresentation cannot be solely attributed to fewer women applying for these specialties. Rather, women are also experiencing lower success rates when matching to specific surgical specialties, both historically and in recent match cycles. Further research and strategies aimed at improving disparities in the surgical residency match should be investigated and appropriate initiatives implemented.

## Supporting information

S1 Checklist Human participants research checklist. (DOCX)

S1 Fig Women Canadian medical graduates from 2003–2022. (DOCX)

S1 Table Proportion of women applicants to surgical specialties from 2003–2022. (DOCX)

S2 Table Proportion of women matriculants to surgical specialties from 2003–2022. (DOCX)
