# Supplementary material for: Gender trends in match rate to surgical specialties in Canada: A retrospective study from 2003–2022
Source: PLoS One. 2024 Apr 10;19(4):e0300207. doi: 10.1371/journal.pone.0300207 (PMC11006131; doi:10.1371/journal.pone.0300207)

**Human Participants Research Checklist**

***Complete the following if your study involved human participants or human participants’ data. These questions should be addressed for prospective and retrospective studies.***

1. Did you obtain ethics approval for this study?
   - If yes, please upload (file type “Other”) the original approval document you received from your ethics committee. If the original document is in another language, please also provide an English translation.

___ Uploaded _X_ N/A

- - If you did not obtain ethical approval, please explain why this was not required below.

We conducted a cross-sectional review of residency match data in Canada from 2013-2022. Data is publicly available online in the public domain, (<https://www.carms.ca/data-reports/r1-data-reports/>), with no expectation of privacy. According to Article 2.2 of TCPS2, Canadian policy framework governing research ethics, research does not require REB review when it relies exclusively on information that is publicly available or in the public domain and the individuals to whom the information refers have no reasonable expectation of privacy. I have attached at the end of the document response from the UBC BREB validating this information. Thank you.

1. If you prospectively recruited human participants for the study – for example, you conducted a clinical trial, distributed questionnaires, or obtained tissues, data or samples for the purposes of this study, please report in the Methods:
   1. the day, month and year of the **start and end** of the recruitment period for this study.
   2. whether participants provided informed consent, and if so, what type was obtained (for instance, written or verbal, and if verbal, how it was documented and witnessed). If your study included minors, state whether you obtained consent from parents or guardians. If the need for consent was waived by the ethics committee, please include this information.

___ Completed _X_ N/A

1. If you are reporting a retrospective study of medical records or archived samples, please report in the Methods section:
2. the day, month and year when the data were accessed for research purposes
3. whether authors had access to information that could identify individual participants during or after data collection

___ Completed _X_ N/A


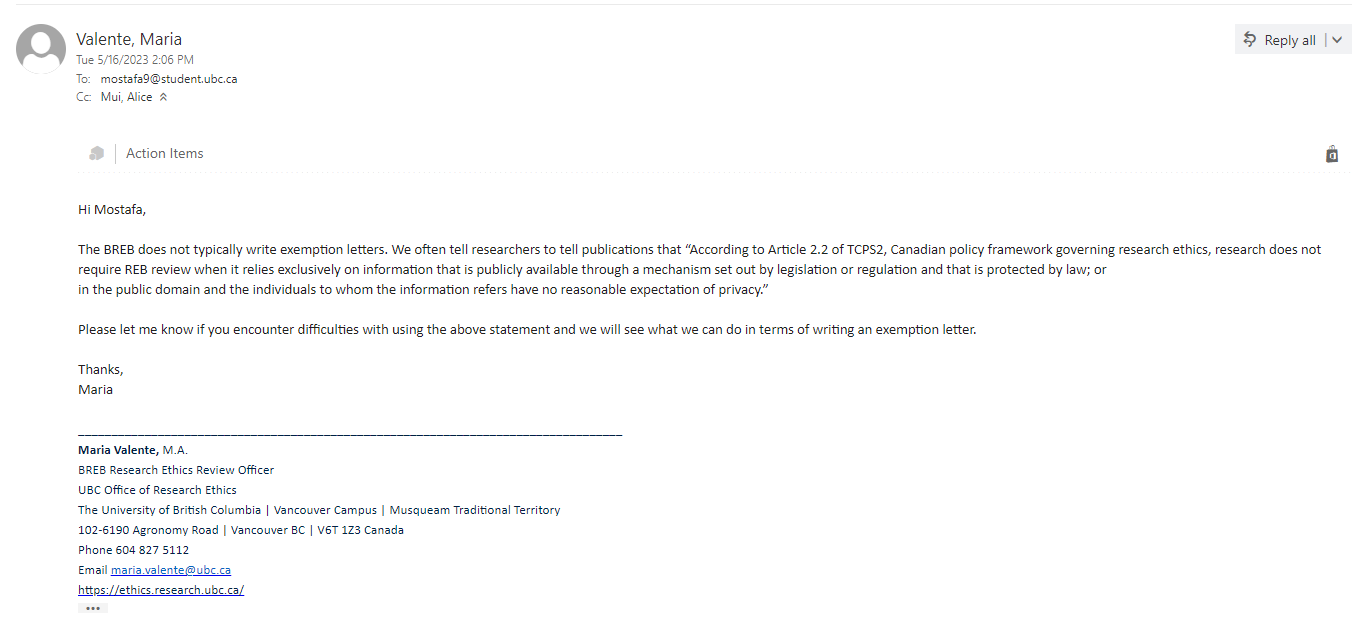

Supplement: S1 Checklist — (DOCX) [file pone.0300207.s001.docx]
