# Supplementary material for: Gender trends in match rate to surgical specialties in Canada: A retrospective study from 2003–2022
Source: PLoS One. 2024 Apr 10;19(4):e0300207. doi: 10.1371/journal.pone.0300207 (PMC11006131; doi:10.1371/journal.pone.0300207)
Supplement: S1 Fig — (DOCX) [file pone.0300207.s002.docx]

**
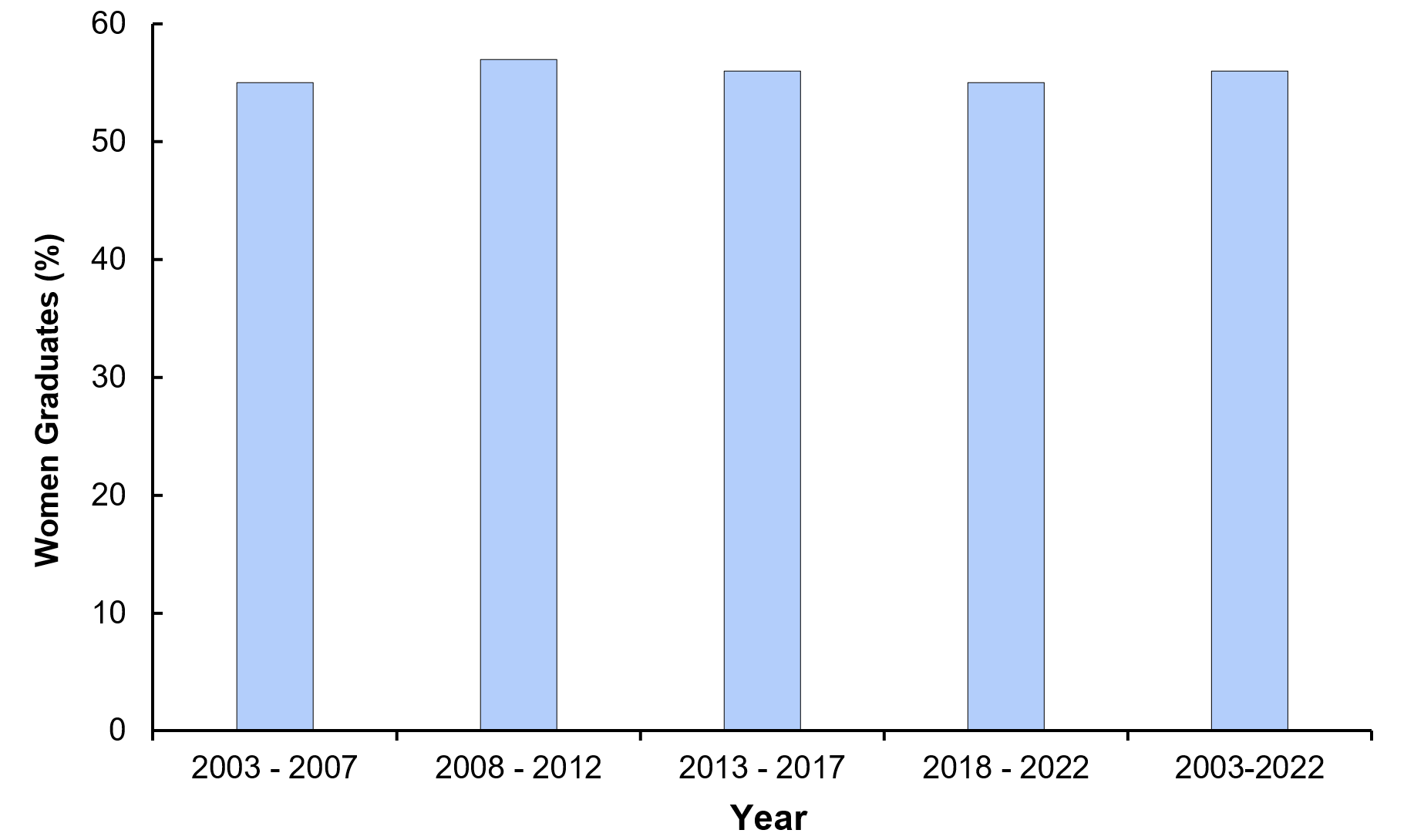
**

**S1 Fig. Women Canadian medical graduates from 2003-2022.** The formula used for percentage of women graduates was (women graduates / [men graduates + women graduates]) *100.
