# Supplementary material for: Gender trends in match rate to surgical specialties in Canada: A retrospective study from 2003–2022
Source: PLoS One. 2024 Apr 10;19(4):e0300207. doi: 10.1371/journal.pone.0300207 (PMC11006131; doi:10.1371/journal.pone.0300207)
Supplement: S1 Table — (DOCX) [file pone.0300207.s003.docx]

**S1 Table. Proportion of women applicants to surgical specialties from 2003-2022.**

| **Specialty** | **2003-2007 (%)** | **2008-2012 (%)** | **2013-2017 (%)** | **2018-2022 (%)** | **2003-2022 (%)** | **p value^†^** |
| --- | --- | --- | --- | --- | --- | --- |
| Cardiac Surgery | 22 | 38 | 35 | 43 | 35 | **0.03** |
| General Surgery | 46 | 49 | 50 | 60 | 52 | **<0.001** |
| Neurosurgery | 15 | 32 | 23 | 30 | 26 | 0.08 |
| Ophthalmology | 36 | 41 | 41 | 38 | 39 | 0.99 |
| Orthopedic Surgery | 23 | 26 | 29 | 35 | 28 | **<0.001** |
| Otolaryngology | 34 | 49 | 44 | 48 | 45 | 0.07 |
| Plastic Surgery | 43 | 51 | 49 | 50 | 49 | 0.29 |
| Urology | 23 | 23 | 30 | 38 | 29 | **<0.001** |
| ObGyn | 88 | 88 | 94 | 89 | 87 | 0.91 |
| Vascular Surgery* | - | - | 32 | 45 | - | 0.15 |
| All Surgery | 45 | 50 | 50 | 55 | 50 | **<0.001** |
| *Data for vascular surgery not available prior to 2012. †P values are from the Cochran-Armitage trend test for proportions. Bold font indicates statistical significance (p<0.05). The formula used for percentage of first choice women applicants was (women applicants / [men applicants + women applicants]) *100. ObGyn: Obstetrics and Gynecology. | | | | | | |
