# Supplementary material for: Gender trends in match rate to surgical specialties in Canada: A retrospective study from 2003–2022
Source: PLoS One. 2024 Apr 10;19(4):e0300207. doi: 10.1371/journal.pone.0300207 (PMC11006131; doi:10.1371/journal.pone.0300207)
Supplement: S2 Table — (DOCX) [file pone.0300207.s004.docx]

**S2 Table. Proportion of women matriculants to surgical specialties from 2003-2022.**

| **Specialty** | **2003-2007 (%)** | **2008-2012 (%)** | **2013-2017 (%)** | **2018-2022 (%)** | **2003-2022 (%)** | **p value^†^** |
| --- | --- | --- | --- | --- | --- | --- |
| Cardiac Surgery | 24 | 36 | 29 | 42 | 33 | 0.15 |
| General Surgery | 47 | 48 | 51 | 60 | 51 | **<0.001** |
| Neurosurgery | 16 | 31 | 22 | 33 | 26 | 0.06 |
| Ophthalmology | 42 | 42 | 42 | 41 | 42 | 0.89 |
| Orthopedic Surgery | 24 | 25 | 27 | 35 | 27 | **0.006** |
| Otolaryngology | 32 | 44 | 42 | 43 | 41 | 0.20 |
| Plastic Surgery | 41 | 47 | 47 | 46 | 46 | 0.52 |
| Urology | 21 | 22 | 25 | 34 | 26 | **<0.001** |
| ObGyn | 87 | 88 | 85 | 87 | 87 | 0.78 |
| Vascular Surgery* | - | - | 26 | 50 | - | **0.03** |
| All Surgery | 46 | 49 | 49 | 54 | 50 | **<0.001** |
| *Data for vascular surgery not available prior to 2012. †P values are from the Cochran-Armitage trend test for proportions. Bold font indicates statistical significance (p<0.05). The formula used for percentage of first choice women matriculants was (women matriculants / [men matriculants + women matriculants]) *100. ObGyn: Obstetrics & Gynecology. | | | | | | |
